# Strontium coating by electrochemical deposition improves implant osseointegration in osteopenic models

**DOI:** 10.3892/etm.2014.2038

**Published:** 2014-10-30

**Authors:** YONGQIANG LIANG, HAOYAN LI, JIANG XU, XIN LI, XINCHANG LI, YUTING YAN, MENGCHUN QI, MIN HU

**Affiliations:** 1 College of Stomatology, Hebei United University, Tangshan, Hebei 063000, P.R. China; 2 Department of Stomatology, Chinese PLA General Hospital, Beijing 100853, P.R. China; 3 Department of Stomatology, People’s Hospital of Tongchuan, Shaanxi 727000, P.R. China

**Keywords:** strontium, coating, implant, osteopenia, osseointegration

## Abstract

Osteopenia, a preclinical state of osteoporosis, restricts the application of adult orthodontic implant anchorage and tooth implantation. Strontium (Sr) is able to promote bone formation and inhibit bone absorption. The aim of the present study was to evaluate a new method for improving the success rate of dental implantation. In this study, an electrochemical deposition (ECD) method was used to prepare a Sr coating on a titanium implant. The coating composition was investigated by energy dispersive X-ray spectroscopy and X-ray diffraction, and the surface morphology of the coating was studied using scanning electron microscopy. A total of 24 Sprague-Dawley rats received bilateral ovariectomy (OVX) and an additional 12 rats underwent a sham surgery. All rats were then implanted in the bilateral tibiae with titanium mini-implants with or without a Sr coating. The results of histological examination and a fluorescence double labeling assay showed strong new bone formation with a wider zone between the double labels, a higher rate of bone mineralization and better osseointegration in the OVX rats that received Sr-coated implants compared with the OVX rats that received uncoated implants. The study indicates that Sr coatings are easily applied by an ECD method, and that Sr coatings have a promoting effect on implant osseointegration in animals with osteopenia.

## Introduction

Osteoporosis is a major factor restricting adult orthodontic implant anchorage and tooth implantation. Previous studies have shown that osteoporosis affects the jawbone and weakens the osseointegration ability of dental implants (1–3).

A reduction of bone mineral density of between 1 and 2.5 standard deviations is defined as osteopenia. Prior to osteoporosis, the human body experiences a pathological process in which bone mineral density is decreased. Osteopenia is a preclinical state of osteoporosis (4). Therefore, osteopenia is considered as a risk factor against the success of implantation.

The conventional treatment method for osteoporosis is drug therapy to prevent bone absorption. However, this modality is associated with adverse effects, drug resistance and long-term risks (5–7). Strontium (Sr) is an essential trace element for human skeletal components. It can promote bone formation and inhibit bone absorption (8). *In vitro* and *in vivo* assays have demonstrated that Sr can promote osteoblast proliferation and inhibit osteoclast proliferation, and *in vitro* experiments have indicated that Sr has a positive role in bone tissue engineering (9).

In the present study, Sr coatings were deposited on the surface of titanium implants by electrochemical deposition (ECD) and the implants were evaluated in an ovariectomized rat model. The aim of the study was to identify a new method for improving the success rate of dental implantation.

## Materials and methods

### Preparation of Sr coating by ECD

Commercial grade 4 unalloyed titanium plates (10×10×2 mm) and custom-made screw-type titanium implants (1.5 mm diameter, 6.5 mm length; 99.8% Ti; National Engineering Research Center in Biomaterials, Sichuan University, Chengdu, China) were used as substrates for ECD. Prior to the deposition of coating, the titanium plates and implants were polished with up to 2,000 grid, immersed into hydrochloric acid and calcium chloride solutions successively, ultrasonically cleaned in acetone for 10 min, rinsed with deionized water and air dried. The ECD experimental setup used for this study was a two-electrode cell configuration, as previously described (10). The working electrode (the coating substrate) was the titanium plate and the counter electrode was a platinum mesh. The distance between the electrodes was 2 cm. The constant voltage was 2.5 V, the temperature was 60°C and the reaction time was 1 h. The electrolytes were NH_4_H_2_PO_4_ 0.036 mol/l, CaCl_2_ + SrCl_2_ 0.06 mol/l and NaCl_2_ 0.1 mol/l, pH 4.5.

### Morphology and characterization of coating

The phase composition of the coating was analyzed by X-ray diffraction (XRD) using a D/MAX2500PC automatic XRD apparatus (Rigaku, Tokyo, Japan). The surface morphology and coating thickness were examined by scanning electron microscopy (SEM) using an S-4800 field emission scanning electron microscope (Hitachi, Tokyo, Japan). SEM was coupled with energy dispersive X-ray spectroscopy using a Noran™ system 7 X-ray microanalysis system (Thermo Fisher, Madison, WI, USA), which was used to determine the elemental composition of the biomimetic coating.

### Animals and ovariectomy (OVX)

A total of 36 adult female Sprague Dawley (SD) rats, aged 3 months, were obtained from the Military Medical Science Academy of the PLA (Beijing, China). The *in vivo* study was approved by the Animal Care and Use Committee of Hebei United University [Tangshan, China; License no. SCXK (Jun 2009–003)]. Bilateral OVX was performed in 24 rats through an incision in the back under general anesthesia with an intraperitoneal injection of 10% chloral hydrate at a dose of 3 ml/kg (Chemical Reagent Co., Shanghai, China), while the remaining animals (12 rats) underwent a sham surgery in which the ovaries were examined and returned to the original position under the same protocol.

### Implantation and animal grouping

Five weeks following OVX or sham surgery, all animals received simultaneous bilateral tibiae implantation using pure screw-shaped titanium implants with an ECD-generated Sr coating. The 12 animals that underwent a sham surgery were designated as the sham group, and the animals that underwent OVX were randomly divided into two groups, with 12 animals/group. One group received implants with a Sr coating (Sr group) and the other received implants without an Sr coating (OVX group).

### Fluorescent labeling

Following implantation, the SD rats were subdermally injected once a day with tetracycline hydrochloride at 30 mg/kg on days 13 and 14 and calcein at 6 mg/kg on days 3 and 4.

### Histological examination

All rats were sacrificed at 4 weeks following implantation. Specimens of tibiae were fixed in 10% neutral buffered formalin for 5 days, dehydrated in increasing gradients of alcohol, and embedded in methylmethacrylate resin. Undecalcified, ground 30-μm sections parallel to the long axis of the implant and vertical to the long axis of tibiae were obtained using an Exakt saw and grinding equipment (Exakt 300; Exact Advanced Technologies, GmbH, Norderstedt, Germany). The sections were stained with toluidine blue. Three sections of each specimen were examined at a magnification of ×100.

Histomorphometric analysis was performed with a semi-automated digitizing image analysis system, comprising a Nikon ECLIPSE E600 stereomicroscope, a computer-coupled Nikon DXM1200 Digital Camera and NIS-Elements F 2.20 image software (Nikon Corporation, Tokyo, Japan). Histomorphometric indices included the implant-bone contact rate (IBCR), defined as the direct implant-bone interface to total implant surface, and the ratio of calcified bone volume to total bone volume (BV/TV) within 2.0 mm of the axis of the implant. In each section, five equally spaced sites of each screw were measured and the mean value of all screws was accepted as the value of the index of the section (11).

### Bone dynamic indices

The distance between double labels (DDL), mineral apposition rate (MAR) and mineralizing surface/bone surface ratio (MS/BS) were calculated. The MAR was determined by measuring the distance between the two fluorescent lines. In each field, five equally distributed sites of each double fluorescent line were measured and the mean of the values in the field was accepted as the value of the field. The measurements were conducted with a confocal microscope (FV1000; Olympus Corporation, Tokyo, Japan). The MS/BS value was calculated as follows: MS/BS (%) = (0.5 single labeled perimeter + double labeled perimeter)/bone perimeter ×100.

### Biomechanical testing

The removal torque of the implants was examined with a method similar to that previously reported (12). Briefly, the specimens were fixed in 10% neutral buffered formalin and embedded in a quadrate metal box with dental plaster. The testing equipment was a force measuring device (Asida DZE-5; Zhengye Electronics Co., Ltd., Dongguan, China) for recording the peak force value in Newtons (N) required to loosen the implant and a specially made wrench that connected the implant and the force measuring device. The implant used was specially designed with a square cap suitable for application of the wrench. The removal torque was calculated by multiplying the peak force value with the distance between the force point and the center of the implant.

### Statistical analysis

Data are expressed as mean ± standard deviation (SD) and statistical analyses were performed using SPSS software, version 12.0 (SPSS, Inc., Chicago, IL, USA). One-way analysis of variance (ANOVA) was used to assess differences between OVX and sham-operated rats. P<0.05 was considered to be indicate a statistically significant difference.

## Results

### Coating XRD analysis

**Figure 1:**
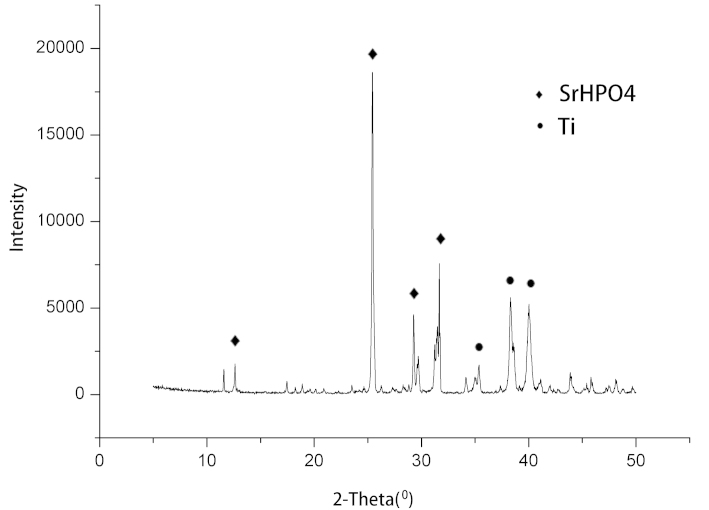
X-ray diffraction analysis of the strontium coating. The results indicate that the coating comprised strontium hydrogen phosphate.

XRD analysis indicated that the coating comprised strontium hydrogen phosphate (SrHPO_4_). The relative intensities of the diffraction peaks for the SrHPO_4_ crystals (102) were higher than those for other crystal face intensities, and revealed a (102) crystal face growth advantage (Fig. 1).

### Analysis of the morphology of the coating surface

**Figure 2:**
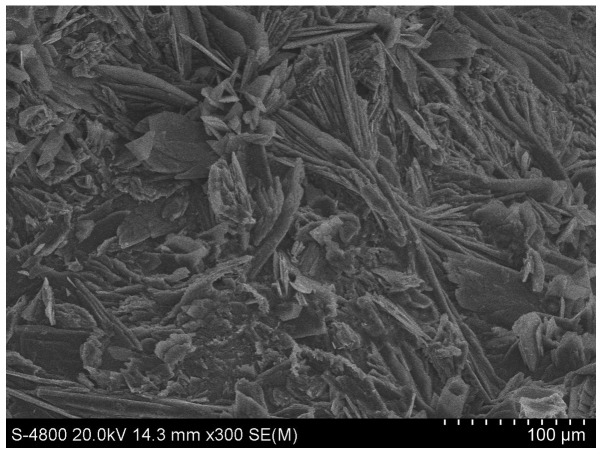
Morphology of the strontium (Sr) coating by scanning electron microscopy. The Sr coating consists of lamellar crystals in aggregated clusters or a petal-like arrangement.

The Sr coating thickness analyzed by SEM was 25 μm. The Sr coating was observed to consist of lamellar crystals in aggregated clusters or a petal-like arrangement (Fig. 2).

### Histological examination

**Figure 3:**
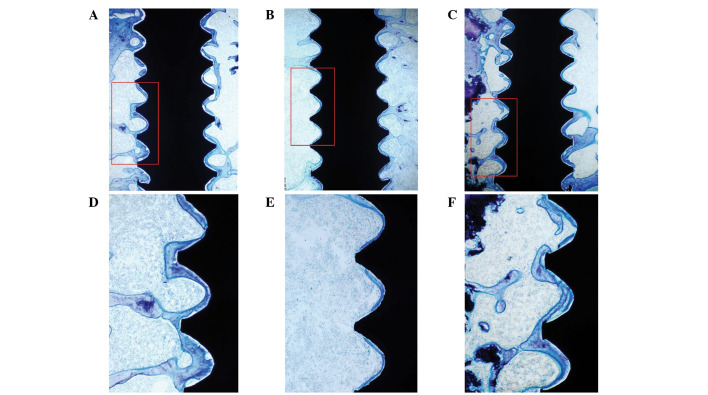
Histological examination of the proximal tibia with implants and bone grafts with toluidine blue staining. Images for (A,D) the sham group, (B,E) OVX group and (C,F) Sr group. The OVX group had the least implant-bone integration and calcified bone, while the Sr-coating group had a marked increase of implant-bone integration and calcified bone. Magnification (A–C), ×40; (D–F), ×100. OVX, ovariectomized; Sr, strontium.

Histological images obtained from undecalcified sections are shown in Fig. 3. In the OVX group, bone formation was diminished with poor bone continuity. By contrast, in the Sr group, new bone formation was evident with new thicker laminar bones. Certain laminar bones were discontinuous. Bone formation was normal in the sham group, with prominent laminar bone. Table I shows various histomorphometric indices, namely the IBCR, BV/TV and values for the thickness of the bone lamellar interface (T). The IBCR, BV/TV and T in the Sr group were significantly higher than those of the OVX group (P<0.01), and the OVX group had the lowest IBCR, BV/TV and T (P<0.05 or P<0.01).

### Fluorescent histology

**Figure 4:**
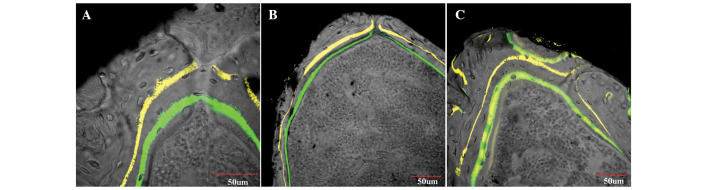
Fluorescent double labeling in the (A) sham, (B) OVX and (C) Sr groups 4 weeks post-implantation as observed by laser scanning confocal microscopy. The OVX group showed the poorest fluorescent labeling, in which the fluorescent intensity was weak, the fluorescent lines were thin and discontinuous, and the distance between the double-labeled line was narrow. Magnfication, ×400. OVX, ovariectomized; Sr, strontium.

Confocal microscopy showed strong fluorescent labeling in the OVX and Sr groups, mainly at the trabecular bone surface and at the interface between the implant and bone (Fig. 4). The sham group showed strong fluorescent intensity, with thick and continuous fluorescently labeled lines, and a wide distance between the two fluorescent lines. However, in the OVX group, the fluorescence intensity was weak, the fluorescently labeled lines were thin and discontinuous, and the distance between the two fluorescent lines was narrow. The appearance of fluorescent labeling was markedly strengthened in the Sr group and was greater than that in the OVX group, although it was not as strong as that in the sham group. The aforementioned changes were also supported by quantitative analysis. The highest bone dynamic indices, specifically DDL, MAR and MS/BS values, were found in the sham group, followed in turn by the Sr and OVX groups (P<0.05 or P<0.01). Significant differences in these indices were also observed between the Sr and OVX groups (P<0.05 or P<0.01). Thus, the Sr group exhibited active bone metabolism (Table II).

### Biomechanical test

The removal torques of the titanium implants in the sham, OVX and Sr groups were 27.94±1.43, 22.04±2.11 and 25.30±1.38 N.cm respectively. The removal torques in the OVX and Sr groups were significantly increased when compared with that of the sham group (P<0.01). The removal torque in the Sr group was higher than that in the OVX group (P<0.01).

## Discussion

Osteoporosis has brought many challenges to clinicians. Osteoporosis and osteopenia restrict orthodontic implant anchorage and tooth implantation in adults. A previous study showed that implant failure in patients with osteoporosis is associated with the thickness of cortical bone (13). In the present study, it was found that new bone formation was increased around the Sr coating, and implant osseointegration was promoted. This suggests that Sr may improve the stability of implants.

Implant osseointegration in patients with osteoporosis is affected by reduced bone mass and bone tissue microstructural changes (3). However, Motohashi *et al* discovered that bone formation and distribution around the implant in rats with osteoporosis are the same as those in normal rats in the early stage of osteoporosis; however the absorption rate of new bone is accelerated in the later stage of osteoporosis (14). The studies conducted by Mori *et al* (15) and Fujimoto *et al* (16) demonstrated that new bone formation around the implant in osteoporotic animals was delayed compared with that in healthy animals. Moreover, osteoporosis affects bone healing around the implant. Due to a reduction of cancellous bone in patients with osteoporosis, these patients require more frequent monitoring of implant stability.

After the implant is implanted in bone, the implant is not able to bear the orthodontic force if healthy bone is lacking around the mini-implant (17). The success of a mini-implant is determined by the stability of the connection between the implant and the bone tissue. Reduced estrogen levels can accelerate the bone metabolic rate and result in decreased quality and density of bone, which affects the initial stability of the implant (18). Osteopenia can develop into osteoporosis (19). Therefore, the timely intervention of osteopenia is necessary for the management of osteoporosis.

Sr reduces bone resorption by inhibiting the proliferation of osteoclasts and improving bone formation by stimulating the proliferation of osteoblasts (20). Sr has also been shown to enhance osteogenic cell replication and the activity of osteoblasts, including the synthesis of bone matrix and the activity of alkaline phosphatase, in addition to reducing osteoclast markers generated in the differentiation of bone marrow cells, inhibiting the differentiation of osteoclasts and reducing the activity of osteoclasts (21,22). Furthermore, Sr stimulates the osteogenic differentiation of bone marrow mesenchymal stem cells and other progenitor cells (23). Studies have confirmed that Sr-doped hydroxyapatite, calcium phosphate, calcium silicate, calcium sulfate, boron bioactive glass and other materials, promote bone tissue reconstruction and new bone formation (24–26). In the present study, Sr significantly enhanced bone formation in ovariectomized rats with Sr-coated implants. This implies that tooth implants with a Sr coating may be useful in osteopenic patients. However, the application of Sr coatings requires further investigation for optimization.

The Sr coatings were deposited on the surfaces of pure titanium implants by ECD. Four weeks after implantation of the Sr-coated implants into the tibiae of the rats with osteopenia, the histomorphometric indices IBCR, BV/TV and T in the Sr group were observed to be significantly higher than those of the OVX group. These changes were also supported by quantitative analysis. The bone dynamic indices DDL, MAR and MS/BS in the Sr group were significantly higher than those of the OVX group. The Sr group exhibited active bone metabolism. The histomorphometric indices and bone dynamic indices confirmed that the presence of Sr at the surface of the implant promoted osteoblast function and inhibited osteoclast function. Ultimately, it promoted osseointegration and increased the removal torque.

In conclusion, Sr coatings are easily made by ECD. They have a promoting effect on implant osseointegration in animals with osteopenia, and provide a new proposition for patients with osteoporosis who require dental implants.

## Figures and Tables

**Table I:** Histomorphometric analysis of bone indices among the three groups (n=12).

Group	IBCR (%)	BV/TV (%)	T (μm)
Sham	62.71±4.60	59.24±5.13	67.01±6.66
OVX	39.34±4.42^a^	42.39±5.48^a^	49.34±4.49^a^
Sr	58.72±3.85^b^,^c^	49.39±7.14^a^,^d^	54.27±6.95^a^,^c^

IBCR, implant-bone contact rate; BV/TV, calcified bone volume/total bone volume; T, thickness of the lamellar bone interface; OVX, ovariectomy. Data presented are the mean ± standard deviation.

a P<0.01 compared with the sham group;

b P<0.05 compared with sham group;

c P<0.01 compared with the OVX group;

d P<0.05 compared with the OVX group.

**Table II:** Dynamic indices of bone metabolism (n=12).

Group	DDL (μm)	MAR (μm/day)	MS/BS
Sham	34.99±3.53	2.72±0.20	0.28±0.05
OVX	20.59±3.13^a^	1.61±0.30^a^	0.16±0.03^a^
Sr	30.41±3.14^a^,^b^	2.30±0.40^b^,^c^	0.20±0.03^c^,^d^

DDL, distance between double labels; MAR, mineral apposition rate; MS/BS, mineralizing surface/bone surface ratio; OVX, ovariectomy. Data presented are the mean ± standard deviation.

a P<0.01 compared with the sham group;

b P<0.01 compared with the OVX group;

c P<0.05 compared with the sham group;

d P<0.01 compared with the OVX group.
